# Exploring the current status of neuroendocrine tumours: a population-based analysis of epidemiology, management and use of resources

**DOI:** 10.1186/s12885-019-6412-8

**Published:** 2019-12-16

**Authors:** Josep Darbà, Alicia Marsà

**Affiliations:** 1 Universitat de Barcelona, Department of Economics, Diagonal 696, 08034 Barcelona, Spain; 2 BCN Health Economics & Outcomes Research S.L., Travessera de Gràcia, 62, 08006 Barcelona, Spain

**Keywords:** Neuroendocrine tumors, Disease management, Population-based study, Economic impact study, Spain

## Abstract

**Background:**

Neuroendocrine tumours (NETs) are rare malignancies characterised by its capacity to synthesise and secrete monoamines, due to its neuroendocrine origin. Its varied locations and symptoms have traditionally been responsible for extended delays in their diagnosis. The interest of this study was to characterise the patient population diagnosed with NETs in Spain and to revise how the disease is managed, together with the hospitalisation costs of these patients.

**Methods:**

The database included records of all patients diagnosed with a NET between 2010 and 2015. Admission records were used to evaluate hospitalisation, disease management data and costs, and single-patient files were used to characterise the population.

**Results:**

Nine Thousand One Hundred Twenty patients were diagnosed with a neuroendocrine tumour between 2010 and 2015, with a 2 fold increase in the diagnosis rate over the study period. 42.25% of the patients were females, while 57.75% were males, and mean diagnosis age was 62.58 years (SD = 14.65). Considering all the registered neuroendocrine neoplasms, 46.86% of the patients had malignant well-differentiated NETs, 32.02% had a malignant poorly differentiated neuroendocrine carcinoma and 42.93% of patients developed metastatic NETs. In addition, 18.59% of patients were diagnosed with benign well-differentiated NETs. The most common tumour sites were the bronchus, lung and other sites, including pancreatic tumours; metastasis was found in the liver and distant lymph nodes. Pancreatic resection was the most common surgical procedure utilised in these patients, summing 19% of total expenses, the injection of an unspecified therapeutic substance (including targeted therapies) was registered in 11.40% of admissions, while chemotherapy was registered in only 6.85% of admissions. The annual healthcare cost of NETs was €15,373,961, corresponding to €9092 per patient.

**Conclusions:**

The implementation of standard diagnosis procedures should be prioritised, with a focus on the pancreas and lung, and taking into account that 42.93% of the patients develop a metastatic tumour. The presence of comorbidities and multimorbidities should be considered in order to develop more efficient disease management protocols.

## Background

Neuroendocrine tumours (NETs) are a type of neoplasms characterised by its capacity to synthesise and secrete monoamines, due to its neuroendocrine origin [1]. NETs are classified according to various features centred on their biological behaviour: grade, differentiation and stage, and by their location [2]. The body-wide distribution of NETs is explained by the extensive presence of neuroendocrine tissue in the human body. Several studies described the lung, pancreas and the gastrointestinal tract as the primary sites to find these tumours, although they can appear in numerous areas generating a variety of symptoms that are diverse, being it complex to associate them directly to the disease [3]. Moreover, functional tumours cause a series of symptoms, known as carcinoid syndrome, primarily affecting the digestive and respiratory systems, the skin and the heart [1].

NETs are considered rare tumours. Data collected over the past 20 years in several European countries and the USA estimated an incidence of 1–5 per 100,000 inhabitants [4], with an increasing tendency over the past years [5].

Their rarity, together with their body-wide presentation and variability, is responsible for the delays in diagnosis that occur frequently. A delay of 52 months on average has been reported in patients with NETs between the first symptoms and diagnosis, and it is common for patients to be examined by several different doctors before receiving the correct diagnosis [6], thus the interest on reviewing the characteristics of affected patients, tumour nature and managing. Altogether, efforts are made to facilitate timely diagnosis, referral and disease management.

Currently, curative treatment depends on the tumour location and generally consists of the resection of the tumour and adjacent tissue that can be accompanied with adjuvant chemotherapy in intermediate and high grade NETs [7–13], other treatments for the control of symptoms are somatostatin analogues or the use of targeted therapies [14]. The therapeutic choice is based on tumour characteristics and most patients require a multidisciplinary care. Still, in some cases tumour behaviour may not correspond with its histology, which leaves patients and professionals with no clear guidelines [1]. Traditionally, a correct and rapid diagnosis of NETs has been challenging due to their indolent history and heterogeneous pathology but with the recent advances in the histopathological characterisation of NETs, diagnosis is becoming accurate and timely, which could be behind their increasing incidence.

The objective of this study was to revise the current state of NETs in Spain, reviewing not only patient and disease characteristics but also disease management protocols and efficiency. NETs economic impact associated with healthcare usage has also been evaluated.

## Methods

### Data extraction

Patient records were extracted from the database under the supervision of the Spanish Ministry of Health, which compiles information from 192 private and 313 public hospitals, covering all Spanish regions. Patients admitted with a neuroendocrine neoplasm in secondary care (inpatient and outpatient) between 2010 and 2015 were identified with the code 209 from the 9th revision of the International Statistical Classification of Diseases and Related Health Problems, Clinical Modification (ICD9-CM). Prior to the extraction, parameters such as health centres and medical history identifiers were re-coded to maintain records anonymised in accordance with the principles of Good Clinical Practice and the Declaration of Helsinki, and thus, ethics committee approval was not required (Ley 14/2007, de 3 de julio, de Investigación biomédica).

### Data analysis

All admission data was used to evaluate information on hospital admission and discharge, days of stay, services that attended the patients, and medical procedures utilised. The extraction of single-patient information was carried out to analyse patient characteristics by eliminating repeated records corresponding to separated admissions, relying on the first admission as the index event.

Within the database, tumours were codified and classified following the ICD9 criteria, firstly according to their behaviour and in malignant well-differentiated NETs, malignant poorly differentiated neuroendocrine carcinomas (NECs), benign well-differentiated NETs and metastatic neuroendocrine tumours (mNETs). Then, tumours were classified according to their origin site. ICD9-CM codes identified patient diagnoses, and medical procedures data was claimed by means of the 9th revision of the International Statistical Classification of Diseases and Related Health Problems, Procedure Classification System (ICD9-PCS). The expenses associated with secondary healthcare were calculated based on the mean costs of medical procedures determined by the Spanish ministry of health, which include the costs related to medical examination, all in-hospital medication, surgery, nutrition, personnel, medical equipment and resources in both inpatient and outpatient admissions. The individual cost of somatostatin analogues and targeted therapy could not be calculated due to the lack of a specific ICD9-PCS code and the costs of prescription drugs were not available in this database.

Data presentation is mainly descriptive. Statistical analyses were performed using Microsoft Excel© Professional Plus 2010 (Microsoft Corporation, Redmond, WA, USA).

## Results

### Patient characteristics

**Fig. 1:**
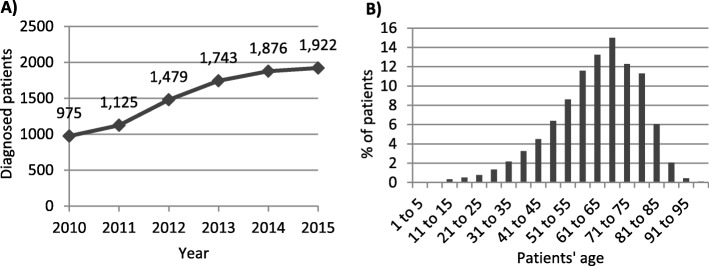
**a** Patients diagnosed with NETs per year in Spain. **b** % of patients diagnosed per age group

Between 2010 and 2015, 9120 patients were diagnosed with a neuroendocrine neoplasm, with an increasing tendency over the years (Fig. 1a). A 2 fold increase was observed in the number of diagnosed patients over a 6 year period. Overall, females represented the 42.25% of these patients, while males were the 57.75%. The different types of neoplasms were diagnosed at a mean age of 62.58 years (SD = 14.65), with virtually no differences between males and females. Tumours were diagnosed in patients ranging from 1 to 104 years, and the analysis per age groups revealed a peak in the group of patients between 66 and 70 years old (Fig. 1b).

The database permitted the analysis of tumour typology, considering that one patient could be diagnosed with distinct tumour types. In total, 12,804 neoplasms were registered; 46.86% of the patients had a malignant NET, 32.02% had a poorly differentiated NEC and 42.93% of patients developed mNETs (Table 1). In addition, 18.59% of patients were diagnosed with benign neuroendocrine tumours.

**Table 1:** Percentage of patients diagnosed per specified tumour type

Tumour classification (ICD9-CM)	% of patients
Malignant neuroendocrine tumours (Well- differentiated), (low grade NET) (carcinoid), (intermediate grade NET) (atypical carcinoid), (high grade NET) (atypical carcinoid).	46.86
209.0 Small intestine	6.95
209.1 Appendix, large intestine, and rectum	5.05
209.2 Other and unspecified sites	34.86
209.20 Unknown primary site	2.46
209.21 Bronchus and lung	17.34
209.22 Thymus	0.14
209.23 Stomach	3.15
209.24 Kidney	0.27
209.25 Foregut, not otherwise specified	0.60
209.26 Midgut, not otherwise specified	2.15
209.27 Hindgut, not otherwise specified	0.29
209.29 Other sites not elsewhere classified	8.46
209.3 Malignant poorly differentiated neuroendocrine carcinomas (High grade NEC not otherwise specified), (small cell lung carcinoma), (small cell uterine carcinoma), (small cell neuroendocrine carcinoma), (large cell neuroendocrine carcinoma), (Merkel cell carcinoma).	32.02
209.30 Malignant poorly differentiated NEC, any site	28.16
209.31 Merkel carcinoma of the face	1.03
209.32 Merkel carcinoma of the scalp and neck	0.31
209.33 Merkel carcinoma of the upper limb	0.56
209.34 Merkel carcinoma of the lower limb	0.68
209.35 Merkel carcinoma of the trunk	0.29
209.36 Merkel carcinoma of the buttock, genitals and others not elsewhere classified	1.00
Benign neuroendocrine tumours (Well- differentiated, (low grade NET) (carcinoid), (intermediate grade NET) (atypical carcinoid).	18.59
209.4 Small intestine	1.23
209.5 Appendix, large intestine, and rectum	2.08
209.6 Other and unspecified sites	15.27
209.60 Unknown primary site	2.37
209.61 Bronchus and lung	6.25
209.62 Thymus	0.04
209.63 Stomach	1.74
209.64 Kidney	0.07
209.65 Foregut, not otherwise specified	0.33
209.66 Midgut, not otherwise specified	1.07
209.67 Hindgut, not otherwise specified	0.60
209.69 Other sites not elsewhere classified	2.80
209.7 Secondary or metastatic neuroendocrine tumours	42.93
209.70 Unspecified site	0.32
209.71 Distant lymph nodes	10.32
209.72 Liver	14.69
209.73 Bone	4.40
209.74 Perineum	3.10
209.75 Secondary Merkel cell carcinoma	0.93
209.79 Other sites not elsewhere classified	9.17

The most common NETs were those of the bronchus and lung and of other sites, which includes pancreatic tumours, mNETs were principally tumours of the liver and distant lymph nodes and for malignant poorly differentiated neuroendocrine tumours site was generally not specified.

In all cases, neuroendocrine neoplasms and metastatic tumours were the hospitalisation motive; yet, secondary diagnoses registered during the admission were also available. Disease comorbidities affecting more than 5% of patients were analysed to further characterise the population (Table 2). In total, 41.62% of the patients had essential hypertension, 18.38% were diagnosed with diabetes mellitus (type I or II), and other disorders that may influence patients treatment were observed, as anaemia, hypercholesterolemia, chronic airway obstruction or renal failure. Additionally, tumour metastases were registered in a large number of patients; presumably attributable to metastatic neuroendocrine tumours affecting principally the liver, lymph nodes and bone.

**Table 2:** Disease comorbidities affecting more than 5% of the patients

Disease comorbidities	% of patients
Unspecified essential hypertension	41.62
Liver metastases	29.42
Diabetes mellitus (type I or II)	18.38
Unspecified hyperlipidaemia	18.33
Lymph nodes metastases	18.23
Respiratory failure and chronic airway obstruction	10.90
Bone metastases	10.69
Renal failure and chronic kidney disease	9.85
Anaemia	9.22
Gastrointestinal secondary neoplasm	8.97
Atrial fibrillation	6.92
Pure hypercholesterolemia	6.33
Diaphragmatic hernia	6.30
Neoplasm related pain	5.48
Obesity	5.37

### Healthcare usage and disease management

Between 2010 and 2015, 11,637 admissions were registered in the database corresponding to patients affected by neuroendocrine neoplasms. Less than one admission was registered per patient per year and mean hospitalisation time was of 11 days. The average readmission rate, understood as a readmission attributable to the same motive within 30 days after discharge, was 16.10%.

To assess disease management several parameters were evaluated, including admission origin, the service to attend the patients, medical procedures, and patients’ condition at discharge. According to the registry, 55.99% of hospital admissions corresponded to scheduled visits, while 43.83% were non-scheduled or urgent. After discharge, the vast majority of patients were discharged into their residence, 83.94%, less than 2% of patients were transferred to other hospitals or extended care facilities, while 12.66% of patients died during the hospitalisation event.

**Fig. 2:**
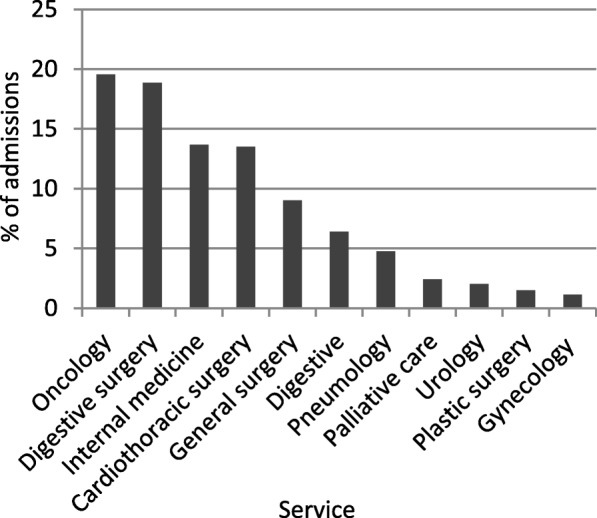
Services to admit patients with NETs in Spain (2010–2015)

Oncology services registered 19.56% of the admissions, followed by admissions into digestive surgery services, 18.86% (Fig. 2).

The analysis of the common medical procedures showed a persistent presence of diagnostic procedures, especially focused on the abdomen and thorax, and a propensity to surgically remove tumours and affected regions (Table 3). Interestingly, the injection of chemotherapy was registered in only 6.85% of total admissions. The injection of other therapeutic substances was registered in 11.40% of admissions, which is likely to include somatostatin analogues and targeted therapies. On the other hand, pulmonary lobectomies and operations were a minority, which suggests that other treatment options were considered for bronchus and lung NETs. A further analysis showed that only 7% of patients with malignant lung NETs were subjected to lobectomies and the same percentage received chemotherapy, while about 10% of patients received another therapeutic substance.

**Table 3:** Management of NETs: Most common medical procedures used to diagnose and treat patients

	Total number	% of admissions
Diagnostic procedures		
Tomography of abdomen or thorax	4979	42.79
Biopsy	3521	30.26
Radiography of abdomen or thorax	2212	19.01
Echography of abdomen or digestive system	1615	13.88
Magnetic resonance imaging (MRI)	695	5.97
Surgery		
Total or partial pancreatectomy	745	6.40
Partial resection of the intestine	498	4.28
Total or partial cholecystectomy	426	3.66
Total or partial splenectomy	338	2.90
Partial hepatectomy	134	1.15
Treatment		
Injection of other therapeutic substance	1327	11.40
Chemotherapy	797	6.85
Radiotherapy	294	2.53
Others		
Antibiotic injection	992	8.52
Palliative care	511	4.39
Steroid injection	364	3.13
Anticoagulant injection	308	2.65
Blood transfusion	182	1.56

### Direct medical cost

The direct cost of NETs associated to the use of healthcare resources in secondary care (inpatient and outpatient) was €15,373,961 per year, corresponding to €9093 per patient per year between 2010 and 2015. Within this cost, surgical procedures, and chemotherapy and radiotherapy administration accounted for most of the expenses (Table 4). The individual cost of somatostatine analogues and targeted therapy could not be assessed. Patients with malignant NETs of the ileum, cecum, thymus, benign NETs of the transverse colon and other sites (including pancreas) displayed the highest costs per patient.

**Table 4:** Annual healthcare usage costs of NETs in Spain including surgical procedures, chemotherapy and radiotherapy

Concept	Annual cost
Total expenses	€15,373,961
Per patient	€1686
Surgery	
Major pancreatic surgery	€2,985,369
Major intestine surgery	€885,885
Cholecystectomy	€874,288
Major hepatic surgery	€536,966
Splenectomy	€424,434
Treatment administration	
Chemotherapy	€361,115
Radiotherapy	€168,855

In addition, 98.39% of the patients were attended under social security financing, and the number of patients under private or mutual care was less than 1%.

## Discussion

### Patient characteristics

The prevalence of NETs in the general population has just recently started being evaluated. These malignancies are overall rare, although research suggest an increase in their diagnosis. A previous retrospective study in Canada displayed a marked increase, of 2.5 fold, of NETs incidence over the course of 15 years [5], comparable to that found herein, probably a consequence of the improvement in their detection. Disease predominance in males has been as well previously described, similarly to the peaking incidence in patients over 65 years [3]. A large number of patients, 78.88%, had a malignant neoplasm, considering NETs and NECs, and the importance of tumours in the bronchus and lung and the gastrointestinal tract support previous evidence that should direct research and diagnostic advances [1, 6].

The presence of comorbidities as essential hypertension and diabetes possibly reflects the ageing nature of the population of study, as for hyperlipidaemia. Alternatively, metabolic disorders have been described in patients treated with certain inhibitors of serine/threonine-specific protein kinases [15]. The present database does not include information regarding patients’ state prior to diagnosis; nonetheless, a previous presence of obesity and metabolic syndrome has been linked with NETs incidence which would require further research [16]. Altogether, comorbidities have a direct influence in the therapeutic choice [2] and should be considered in the principal medical protocols.

### Healthcare usage and disease management

The use of molecular tumour markers was not evaluable via this database, although, as expected, diagnostic imaging procedures were preferred in patients with suspected NETs, favouring tomography or MRI techniques [2]. Secondarily, biopsies were employed. In terms of treatment, surgery appeared as the primary therapeutic option, and pancreatectomies were predominant, which indicates a prevalence of pancreatic tumours in these patients although the exact number could not be determined due to the lack of a specific code for this site.

Less than one new admission per year was registered after the diagnosis of a NET, yet the readmission rate was 16.10%. Previous follow-up studies suggest that readmissions are likely related to postoperative complications after pancreatectomy [17], and patients’ admissions into oncology or surgery services are in line with cancer management protocols. The percentage of registered chemotherapy sessions was surprisingly low; it is possible that its importance was diluted in total admission data, nonetheless limitations due to the database characteristics cannot be ruled out. Moreover, the database characteristics impeded a direct analysis of the use of targeted therapies and somatostatin analogues which should be addressed in further studies; currently, the use of these therapies is codified as the injection of another therapeutic substance not specified in another code.

In terms of prognosis and survival, mortality rates vary enormously with tumour characteristics. In patients with pancreatic NETs 5-year survival rates of 53.9% have been registered [18], although previous follow-up studies of patients after aggressive resection suggest an increase in the 5-year survival up to 80% [19]. To infer survival in the population of study, discharge data was evaluated. Herein patients’ mortality could only be measured when it was registered during hospitalisation; yet, in-hospital mortality reached the 12.66%.

### Direct medical cost

The healthcare usage cost associated with NETs is largely unknown, and it originates rising interest given the increased number of diagnosis in the past years. Herein, the €9093 per patient per year are the sum of the direct costs of hospital admissions (inpatient and outpatient care) including all medical procedures, with almost 3 million euros destined to pancreatic surgery only, which represents 19% of total annual secondary care expenses for the disease. A preceding study in the United States suggested that those patients with pancreatic tumours account for the highest costs [20]. Similarly, data obtained from a cancer registry in Canada supports this statement [21]. Our data suggests that this cost could be linked to the resection of benign tumours. Primary care costs and pharmaceutical expenses should be considered in further analysis to calculate the total medical costs of NETs. In addition, Hallet and colleagues predicted an increase of treatment costs over time due to drug maintenance therapy in patients with grastrointestinal NETs, obtaining an inpatient care cost that reached the €9469 per patient at the time of diagnosis [22].

Presumably, patients’ old age increases overall costs as it raises the number of chronic diseases that are diagnosed in these patients, given the augmented needs and high costs that have been associated with multimorbidities including diabetes, anaemia and hypertension [22, 23].

A series of factors limit the conclusions were subjected to a series of limitations that derive from the database characteristics. Data is codified via ICD9 codes that not correlate with the most recent NET classification criteria as expressed by the World Health Organisation. The lack of specific ICD9 codes to identify somatostatin analogues and targeted therapies impedes the quantification of these drug-related costs individually. Equally, primary care and pharmaceutical data was not available. This study provides novel data on the minimal existing literature regarding the management of NETs and their costs; nevertheless, further research will be required to determine the total burden that NETs represent for the Spanish National Healthcare System.

## Conclusions

A 2 fold increase was measured in the number of NETs diagnoses between 2010 and 2015. All data suggest that tumours primarily affect the pancreas, lung and the gastrointestinal system, and that tumours are mainly resected, which supposes an important portion of economic costs. Herein, the use of adjuvant chemotherapy was not generalised.

Standard diagnosis procedures should be implemented, with a focus on the pancreas and lung, and taking into account that at least 78.88% of patients ultimately develop a malignant tumour. Disease management protocols should consider this data, together with disease comorbidities, in order to develop more efficient treatment protocols.

## Abbreviations

ICD9: 9th revision of the International Statistical Classification of Diseases and Related Health Problems
MRI: Magnetic resonance imaging
NET: Neuroendocrine tumour
